# *PIK3CA* Mutations as a Molecular Target for Hormone Receptor-Positive, HER2-Negative Metastatic Breast Cancer

**DOI:** 10.3389/fonc.2021.644737

**Published:** 2021-03-25

**Authors:** Nicola Fusco, Umberto Malapelle, Matteo Fassan, Caterina Marchiò, Simonetta Buglioni, Simonetta Zupo, Carmen Criscitiello, Paolo Vigneri, Angelo Paolo Dei Tos, Eugenio Maiorano, Giuseppe Viale

**Affiliations:** ^1^Division of Pathology, IEO, European Institute of Oncology IRCCS, Milan, Italy; ^2^Department of Oncology and Hemato-Oncology, University of Milan, Milan, Italy; ^3^Department of Public Health, University Federico II, Naples, Italy; ^4^Department of Pathology, Padua University Hospital, Padua, Italy; ^5^Department of Medicine (DIMED), University of Padua, Padua, Italy; ^6^Division of Pathology, Candiolo Cancer Institute FPO-IRCCS, Candiolo, Italy; ^7^Department of Medical Sciences, University of Turin, Turin, Italy; ^8^Division of Pathology and Cytopathology, Regina Elena National Cancer Institute IRCCS, Rome, Italy; ^9^Department of Pathology, IRCCS Ospedale Policlinico San Martino, Genova, Italy; ^10^Division of Early Drug Development for Innovative Therapies, IEO, European Institute of Oncology IRCCS, Milan, Italy; ^11^Experimental Oncology and Hematology Center, A.O.U. Policlinico “G. Rodolico - S. Marco”, Catania, Italy; ^12^Department of Clinical and Experimental Medicine, University of Catania, Catania, Italy; ^13^Department of Emergency and Organ Transplantation, University of Bari Aldo Moro, Bari, Italy

**Keywords:** breast cancer, biomarkers, PIK3CA, targeted therapy, HR+/HER2-, RT-PCR, next-generation sequencing, liquid biopsy

## Abstract

Despite the significant achievements in the diagnosis and treatment of metastatic breast cancer (MBC), this condition remains substantially an incurable disease. In recent years, several clinical studies have aimed to identify novel molecular targets, therapeutic strategies, and predictive biomarkers to improve the outcome of women with MBC. Overall, ~40% of hormone receptor (HR)^+^/HER2^−^ MBC cases harbor alterations affecting the (PI3K)/Akt/mammalian target of rapamycin (mTOR) pathway. This pathway is a major target in oncogenesis, as it regulates growth, proliferation, cell survival, and angiogenesis. Lately, the pharmacologic targeting of PIK3CA in HR^+^/HER2^−^ MBC has shown significant benefits after the occurrence of endocrine therapy resistance. The orally available α-selective PIK3CA inhibitor, alpelisib, has been approved in this setting. To perform an optimal patients' selection for this drug, it is crucial to adopt a tailored methodology. Clinically relevant *PIK3CA* alterations may be detected in several biospecimens (e.g. tissue samples and liquid biopsy) using different techniques (e.g. real-time PCR and next-generation sequencing). In this study, we provide an overview of the role of PIK3CA in breast cancer and of the characterization of its mutational status for appropriate clinical management.

## Background

### Clinical Scenario

Breast cancer is the most common form of tumor and is the leading cause of cancer-related deaths in the female population worldwide, with a continuous rise in numbers (1). Despite advancements in the treatment of these patients, metastatic breast cancer (MBC) substantially remains an incurable disease (2). Difficulties related to the clinical management of these patients include prolongation of overall survival (OS), control of increasingly severe symptoms, and preservation of the health-related quality of life (3, 4). The currently available therapeutic options for MBC are chemotherapy, endocrine therapy (ET), targeted therapies, and immunotherapy (5). Regrettably, the median OS of MBC has steadily been ~3 years, with a 5-year survival rate of <26% (6).

Two-thirds of breast cancer cases express hormone receptors (HR) and lack HER2 overexpression and/or amplification (7, 8). For these patients, ET is the foremost medical treatment (9). The combination of ET with cyclin-dependent kinases (CDK)4/6 inhibitors (i.e., abemaciclib, palbociclib, and ribociclib) demonstrated significant survival benefits and is now considered the gold standard for HR^+^/HER2^−^ MBC (10, 11). A significant percentage of patients, however, eventually develop ET resistance due to several mechanisms, including the dysregulation of phosphoinositide 3 kinase (PI3K)/Akt/mammalian target of rapamycin (mTOR) signaling (12). Approximately 40% of HR^+^/HER2^−^ MBC cases show hyperactivation of this pathway, which has now become a therapeutic target for the treatment of breast cancer (13).

### Targeting the PI3K Pathway in HR^+^/HER2^–^ Advanced Breast Cancer

In the past decade, several translational research studies and clinical trials aimed to define the efficacy of targeting the PI3K pathway in breast cancer. However, the development of PI3K inhibitors has been historically troubled by toxicity, suboptimal activity, and the lack of reliable diagnostic strategies for an accurate selection of candidate patients (14). In the BOLERO-2 landmark study, the combination of ET with the mTOR inhibitor, everolimus, has been evaluated in postmenopausal women with ET-refractory HR^+^/HER2^−^ MBC (15). In phase III clinical trial, 724 patients who have progressed after ET with aromatase inhibitors (AIs) were randomized to receive either exemestane plus everolimus or placebo plus exemestane. The study met its primary endpoint by showing a significant progression-free survival (PFS) benefit for the everolimus arm (HR 0.43; 95% CI: 0.35–0.54; *p* < 0.001), with an assessment from a local investigator. The blinded independent central review revealed a median PFS of 10.6 months in the experimental arm against a median PFS of 4.1 months in the control arm (HR 0.36; 95% CI: 0.27–0.47; *p* < 0.001). The OS was similar in both groups (median of 31 months in the everolimus group vs. 26.6 months in the placebo group, HR 0.89; 95% CI: 0.73–1.10; *p* = 0.14) (16). Given the substantial benefits observed, everolimus is now approved for use in HR^+^/HER2^−^ MBC upon progression after AI therapy; however, no predictive biomarkers are available to date.

Buparlisib is a pan-PI3K inhibitor that has been evaluated in the BELLE-2 study as monotherapy or has been used in combination with ET and/or anti-HER2 therapy or cytotoxics in *PIK3CA*-mutant breast cancer cases (17). Despite the very low activity/toxicity ratio of this combination for the integration of buparlisib in clinical practice, the results of the BELLE-2 trial suggested that PI3K inhibition along with ET might provide clinically meaningful benefits to postmenopausal women with ET-resistant, HR^+^/HER2^−^ MBC harboring *PIK3CA* mutations (18). Thereafter, targeting the PI3K/Akt/mTOR axis with more specific inhibitors has become the subject of great scientific efforts (19).

The orally available α-selective PIK3CA inhibitor, alpelisib, was the first PI3Kα inhibitor to demonstrate improvement in PFS in HR^+^/HER2^−^ MBC cases with activating *PIK3CA* mutations (20). The SOLAR-1 trial included patients who were resistant to ET, with disease recurrence/progression on or after prior AI therapy (21). That study had two cohorts based on the *PIK3CA* mutational status. In each cohort, patients were randomized to receive alpelisib plus fulvestrant or placebo plus fulvestrant at the ratio of 1:1. The primary endpoint of the trial was PFS in the *PIK3CA*-mutated cohort. With a median follow-up of 20 months, the median PFS for the *PIK3CA*-mutated cohort was almost double with the addition of alpelisib (11.0 vs. 5.7 months, HR 0.65; 95% CI: 0.50–0.85; *p* < 0.001). Significant benefits were also demonstrated in terms of the overall response rate (26.6% [95% CI: 20.1–34.0] vs. 12.8% [95% CI: 8.2–18.7]) and the clinical benefit rate (61.5% [95% CI: 53.8–68.9] vs. 45.3% [95% CI: 37.8–53.1]). The OS in the *PIK3CA*-mutant cohort was a key secondary endpoint. In the final OS analysis for the *PIK3CA*-mutated cohort, OS did not cross the pre-specified O'Brien-Fleming efficacy boundary (one-sided *p* ≤ 0.0161), although median OS was prolonged by a clinically relevant figure of 7.9 months for patients in the alpelisib plus fulvestrant arm; no benefit was seen in the *PIK3CA* non-mutant cohort (22). Additionally, α-selective mutant-degrading PI3K inhibitors (e.g., GDC-0077) are currently under investigation in breast cancer (NCT03006172, NCT04191499).

## Biology and Actionability of *PIK3CA* Mutations

### PI3K/Akt/mTOR Pathway Signaling in Breast Cancer

**Figure 1:**
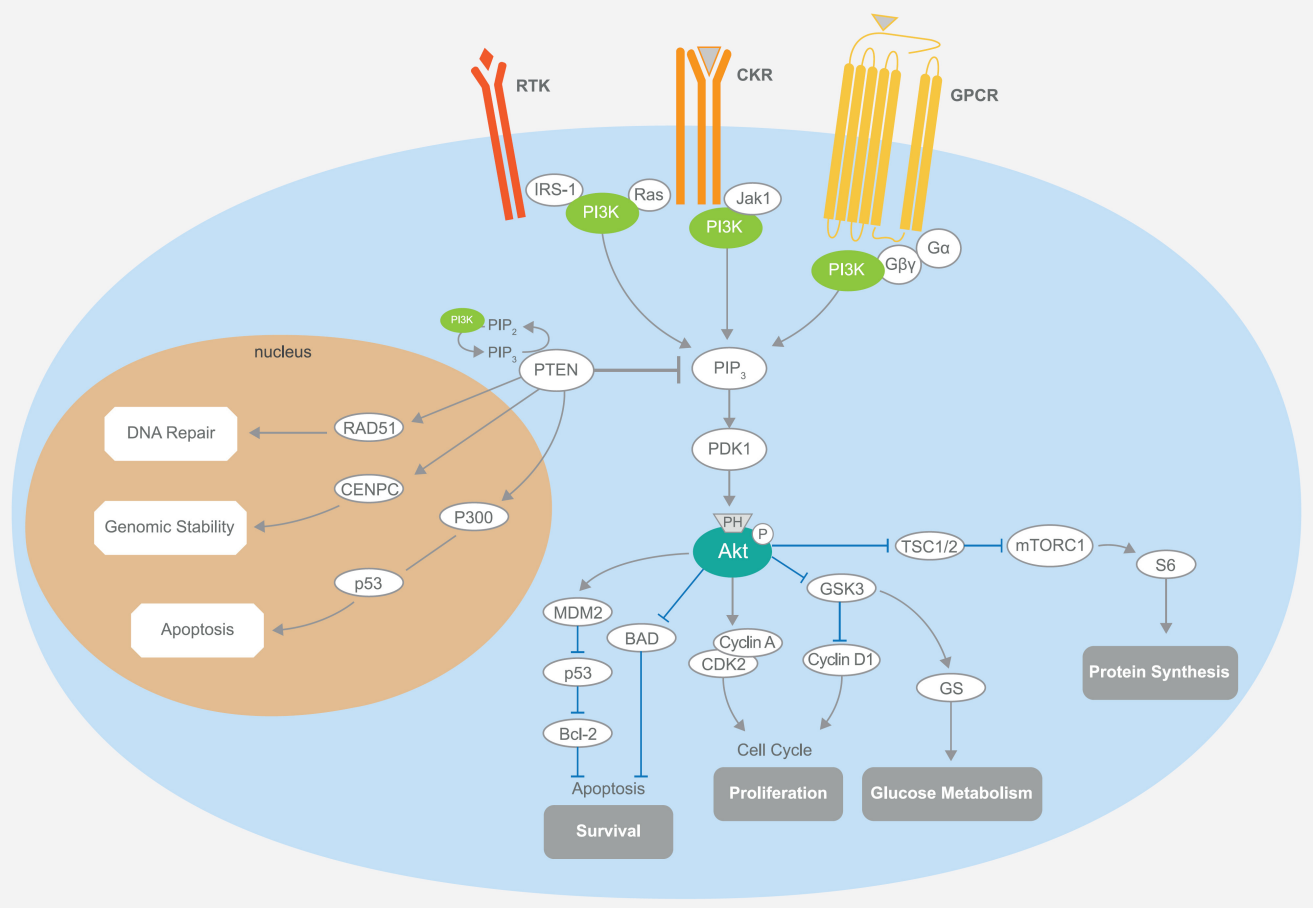
Schematic representation of the PI3K/Akt/mTOR signaling and its main components. The PI3K pathway regulates diverse cellular processes, including protein synthesis, cell survival, proliferation, glucose metabolism, apoptosis, DNA repair, and genome stability. Akt-mediated phosphorylation inhibits the activity of the TSC1–TSC2 complex, also known as hamartin-tuberin. This is a critical step for the negative regulation of mTORC1, whose activity controls anabolic processes. Another important downregulation of Akt phosphorylation is toward BAD, while MDM2 activity is enhanced, promoting the degradation of the tumor-suppressor p53, which also plays a part in the P300-mediated cell apoptosis. Cell cycle regulation occurs through the stimulation of cyclins A and D and the inhibition of GSK3. The latter event is also responsible for increased glucose metabolism. PTEN is intimately involved in the regulation of these mechanisms through its substrate PIP3. Notably, the activity of PTEN in the cell nucleus that leads to cell survival control is related to the upregulation of key mediators, such as RAD51, CDNPC, and P300. RTK, receptor tyrosine kinase; CKR, chemokine receptor; GPCR, G protein-coupled receptor; IRS-1, insulin receptor substrate 1; PI3K, phosphatidylinositol-3 kinase; JAK1, Janus kinase 1; PIP3, phosphatidylinositol-3,4,5-trisphosphate; PDK1, pyruvate dehydrogenase lipoamide kinase isozyme 1; TSC, tuberous sclerosis complex; mTORC1, mammalian target of rapamycin complex 1; MDM2, mouse double minute 2 homolog; BAD, BCL2 associated agonist of cell death; GSK3, glycogen synthase kinase-3; CDK2, cyclin-dependent kinase 2; CDNPC, centromere protein C.

Phosphatidylinositol-3 kinases are heterodimeric lipid kinases characterized by regulatory (p85) and catalytic (p110) subunits, with the former subunit inhibiting the latter in quiescent cells (23). PI3Ks are involved in several cellular processes, such as protein synthesis, cell proliferation and survival, glucose homeostasis, and DNA repair (24, 25). The interaction of the catalytic subunit with the phosphotyrosine residues of activated growth factor receptors or adaptor proteins (e.g., RAS proteins) is critical to elicit PI3K activation (26). As a consequence, the membrane lipid phosphatidylinositol-4,5-bisphosphate (PIP2) is converted to phosphatidylinositol-3,4,5-trisphosphate (PIP3) (27). PIP3 directly activates Akt and other proteins harboring the PIP3-binding pleckstrin-homology (PH) domains (12, 28). Upon complete activation, Akt induces multiple downstream cytosolic and nuclear effectors. This mechanism, considered as the “core” of cell survival and cell cycle progression, is turned off by several phosphatases (PTEN, TSC1, TSC2, and LKB1) that dephosphorylate mTORC1 and PIP3 (29–31) (Figure 1).

Three classes of PI3Ks enzyme isoforms can be distinguished based on their coding genes, chemical structures, and substrate specificity/preferences (23). Mutations involving class IA genes, namely the *PIK3CA* alpha isoform which encodes p110α, are frequently associated with cancer development, progression, and drug resistance in many types of solid tumors, including HR^+^/HER2^−^ breast cancer (32–36). Thus, in May 2019, the U.S. Food and Drug Administration (FDA) approved a α-selective PIK3CA inhibitor, alpelisib (BYL719; Novartis Pharma AG), in combination with fulvestrant for the treatment of postmenopausal patients diagnosed with HR^+^/HER2^−^ *PIK3CA*-mutated, advanced breast cancer or MBC, following the progression on or after an endocrine-based regimen (37, 38). Several other compounds with selective activity against different PI3K isoforms (α, β, γ, and δ) or acting as pan-PI3K inhibitors (on all class I isoforms) have also been investigated. However, their clinical value in terms of effectiveness and toxicity profile remains controversial (39). Of note are the mutations in the PI3K regulatory subunit 1 (*PIK3R1*), which inhibits the α subunit, that can be observed in ~3% of patients with breast cancer (39, 40).

**Figure 2:**
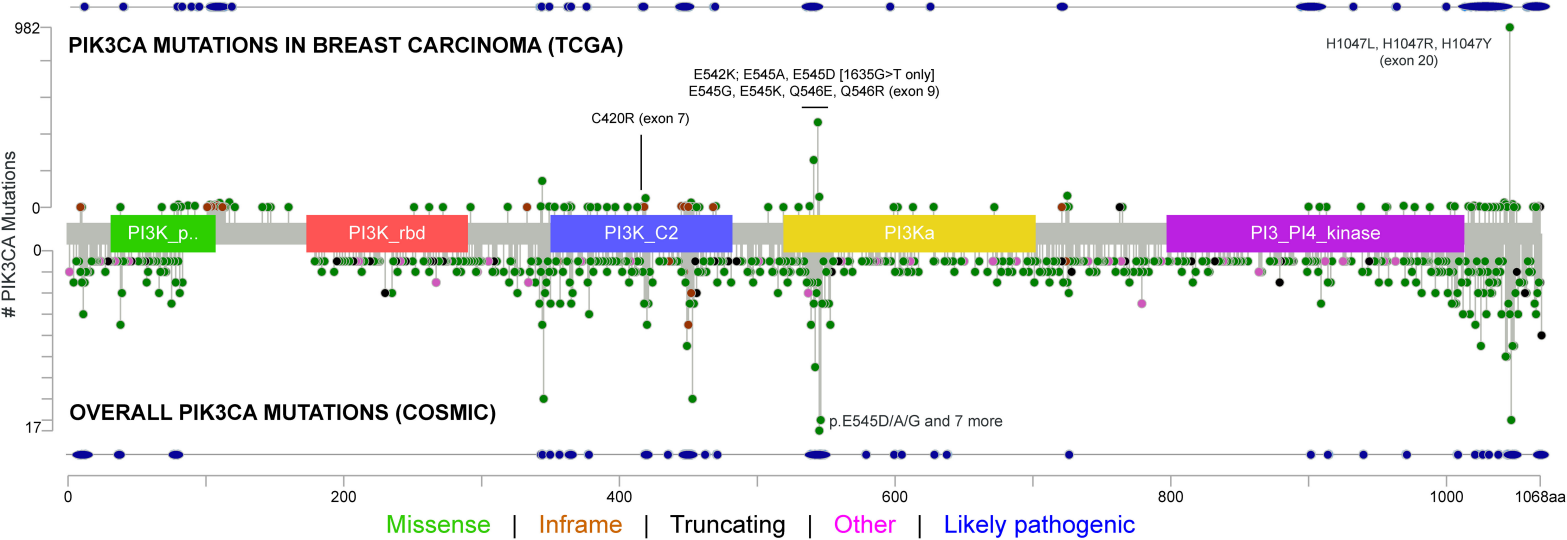
Type of mutations, frequency, and affected PIK3CA domains across breast cancers from The Cancer Genome Atlas (TCGA) Network and selected solid tumors from the Catalog Of Somatic Mutations In Cancer (COSMIC) datasets, including the mutations and exons covered by the FDA-approved the RT-PCR test. The tumor types included in this analysis are non-small cell lung cancer (i.e., squamous cell and adenocarcinoma), esophageal cancer, stomach cancer, colorectal cancer, cholangiocarcinoma, pancreatic cancer, liver cancer, bladder cancer, prostate adenocarcinoma, uterine cancer (i.e., endometrioid, serous, and carcinosarcoma), ovarian cancer, and invasive breast cancer. The types of mutations and their likely pathogenicity are color-coded based on the legend at the bottom.

Different aberrations in the PI3K signaling pathway, such as PI3K mutation/amplification, loss/mutation of the phosphatase and tensin homolog, Akt overexpression/overactivation, and modulation of tuberous sclerosis protein 1 and 2 tumor suppressors, can be often observed in HR^+^ breast cancers. Different *PIK3CA* mutations affecting multiple domains of the protein have been shown in Figure 2 and Supplementary Table 1. In patients with breast cancer, the most common alterations clustered in the helical (exon 9 p. E545K and p.E542K) or the kinase (exon 20 p.H1047R) domains (41). Martínez-Sáez et al. confirmed the clustering, reporting that the most common *PIK3CA* alterations (69% of the total identified) affect exon 20 (p.H1047R in 35% and p.H1047L in 4% of patients) and exon 9 (p.E545K in 17% and p.E542K in 11% of patients) (42). All these alterations respond to alpelisib (20, 38, 39, 42). The fifth most frequently identified alteration was in exon 4 (p.N345K). Despite its likely pathogenicity, as reported in the COSMIC and OncoKB databases (43, 44), only pre-clinical studies have so far demonstrated the sensitivity of this mutation to PI3K inhibitors (45, 46). Another relevant *PIK3CA* alteration is the p.E726K substitution in exon 13, accounting for about 2.5% of mutated cases (42). Despite a low oncogenic activity *per se*, this mutation is frequently associated with other pathogenic *PIK3CA* sequence alterations, amplifying PI3K activity (47). Notably, although rare (0.7% of the analyzed samples), the *PIK3CA* exon 20 p.G1049R mutation is likely pathogenic, and pre-clinical models showed that this mutation, similar to p.E542K, leads to an increased alpelisib sensitivity (46). Similar sensitivity results, in pre-clinical studies, have been obtained for the exon 9 p.Q546K (0.8%) alteration (48). Finally, among other less frequent *PIK3CA* mutations, exon 7 p.C420R (1.9%), exon 9 p.Q546R (1.1%), p.E545A (0.5%), and p.E545G (0.5%) demonstrated responsiveness to alpelisib (21). It is worth mentioning that there is recent evidence to demonstrate that double cis-regulatory *PIK3CA* mutations are related to increased activation of the PI3K signaling compared to single mutants (47). One of the possible biological mechanisms underpinning this hyperactivation could be due to increased membrane binding and p85α disinhibition. Interestingly, tumors showing multiple *PIK3CA* mutations are markedly sensitive to PI3Kα inhibitors (47).

### Molecular Biology Techniques to Detect *PIK3CA* Mutations

#### Direct Sequencing

Direct or Sanger sequencing is the gold standard for the mutational assessment of different biomarkers due to its reliability, availability, reagent affordability, and relatively low costs. Despite these advantages, low sensitivity remains its main limitation (49). Arsenic and colleagues compared the results obtained by Sanger sequencing and next-generation sequencing (NGS) for *PIK3CA* hotspot mutations in exons 9 and 20 in 184 breast cancer samples, reporting a concordance rate of 98.4% (*n* = 52 mutated cases) (50). The three mutated tumors identified by NGS and missed by Sanger sequencing displayed a low (i.e., < 10%) allelic frequency. In another study, Sanger sequencing showed a lower performance rate in the detection of *PIK3CA*-mutated cases compared to multiplex PCR-mass spectroscopy (concordance rate 69%, 35/51 detected cases) and locked nucleic acid (LNA)-PCR (concordance rate 64%, 36/55 detected cases) (51).

#### Real-Time PCR

Real-time PCR (RT-PCR) is widely used to identify mutations in known genomic regions (52). Alvarez-Garcia et al. (53) proposed a simple, fast, and inexpensive diagnostic tool to determine the *PIK3CA* mutational status in patients with breast cancer based on the standard SYBR Green RT-PCR approach. Their findings support the use of RT-PCR to detect *PIK3CA* exon 20 p.H1047R and exon 9 p.E545K hotspot mutations with sensitivities of 5 and 10%, respectively. In the study performing 46 formalin-fixed and paraffin-embedded breast carcinoma specimens, Lambert et al. (54) compared three different RT-PCR approaches, namely the cobas® PIK3CA Mutation Test (Roche Diagnostics, Meylan, France), the PCR amplification-refractory mutation system (ARMS) Scorpion, and the High-Resolution Melting (HRM) PCR assay. Overall, 38% (*n* = 17), 28% (*n* = 13), and 41% (*n* = 19) of the tested samples showed a *PIK3CA* mutation with cobas®, ARMS, and HRM assays, respectively. The highest concordance rate was observed between cobas® and HRM [*k* = 0.95 (0.86; 1)], followed by cobas® and ARMS [*k* = 0.75 (0.55; 0.95)] and HRM and ARMS [*k* = 0.72 (0.51; 0.92)] (54). In a retrospective series, Harlè et al. (55) analyzed 102 breast cancer samples using both HRM and ARMS-PCR for *PIK3CA* exon 9 and exon 20 mutations. Taken together, 27.5 and 22.5% of *PIK3CA* substitutions were reported with PCR-HRM and PCR-ARMS, respectively. Based on these results, we proposed a combined approach with PCR-HRM and PCR-ARMS to better cope with the cost of routine *PIK3CA* mutation identification for invasive breast cancer. In line with these findings, a *PIK3CA* mutation rate of 33.4% has been reported by Cizkova et al. (56) using RT-PCR for exons 9 and 20. In a large, unselected cohort of 1,281 female patients with HR^+^ MBC, Chan et al. (57) adopted the FDA-cleared RT-PCR test (therascreen® PIK3CA RGQ PCR Kit [QIAGEN GmbH, Hilden, Germany]) that covers eleven mutations (exon 7 p.C420R; exon 9 p.E542K, p.E545A, p.E545D, p.E545G, p.E545K, p.Q546E, p.Q546R; and exon 20 p.H1047L, p.H1047R, p.H1047Y) in the *PIK3CA* gene, reporting a 37.5% mutation rate. The same technical approach was also employed in a large analysis by Martínez-Sáez et al. (42) that correctly detected the majority (72%) of the *PIK3CA* mutations of the analyzed samples.

#### Next-Generation Sequencing

The massively parallel sequencing technology, also referred to as NGS, has revolutionized the clinical management of patients with cancer (58). Despite different platforms being commercially available, the NGS workflow follows the same four sequential phases (i.e., library generation, clonal amplification, massively parallel sequencing, and data analysis) (59). Hempel et al. (13) adopted a broad NGS panel to analyze 41 MBC samples and detected *PIK3CA* mutations in 34% (*n* = 14) of them. In another study including patients with ET-resistant (*n* = 15) and ET-sensitive (*n* = 9) breast cancer cases, the same authors were able to identify *PIK3CA* alterations in 55% (*n* = 8) and 33% (*n* = 3) of the cases, respectively (60). Specifically, ET-resistant tumors displayed three pathogenic variants in the kinase domain, three pathogenic variants in the helical domain, and two variants of unknown significance, whereas ET-sensitive tumors presented two pathogenic variants in the kinase domain and one pathogenic variant in the helical domain. In a recent SAFIR02 study, 22% of the total population and 28% of patients with HR^+^ breast cancer featured a *PIK3CA* mutation (61). Using a broad NGS panel targeting 1,021 genes on 193 MBC samples, Tang et al. (62) detected 36 (18.7%) mutations in the kinase domain and 26 (13.5%) substitutions in the helical domain with 10 (5.2%) additional alterations distributed in the remaining *PIK3CA* sequence. One of the main advantages of NGS is that it allows for the identification of multiple mutations simultaneously, avoiding the need to perform sequential individual tests.

#### Analysis of Liquid Biopsy Samples

Liquid biopsy using blood components to assess *PIK3CA* mutations in circulating tumor DNA (ctDNA) of patients with breast cancer has been reported by different studies (39). Multiple technologies have been employed to assess breast cancer ctDNA with high sensitivity and specificity, leading to assays that have been useful in clinical trials and are entering clinical practice (63). Using an ARMS allele-specific PCR and Scorpion probes, Board et al. (64) were able to detect *PIK3CA* mutations in the vast majority (80%) of ctDNA samples from *PIK3CA*-mutated MBC but not in early breast cancer. Digital droplet PCR has been proposed as a more sensitive approach in the non-metastatic setting (65). The secondary endpoint in the SOLAR-1 study was to assess PFS according to the level of ctDNA (21). This trial confirmed that treatment with alpelisib and fulvestrant provided an extension of PFS for patients with *PIK3CA*-mutated disease based on ctDNA analysis. In particular, there was a 45% reduction of risk in PFS for patients with ctDNA *PIK3CA* mutations (HR 0.55; 95% CI: 0.39–0.79; *n* = 186) and a 20% reduction of risk in PFS for those without the mutations (HR 0.80; 95% CI: 0.60–1.06; *n* = 363). The increased magnitude of the PFS benefit for the alpelisib plus fulvestrant arm compared to the placebo plus fulvestrant arm was maintained at subgroup analyses, further highlighting the clinical value of liquid biopsy (66). Comparative analysis of *PIK3CA* mutations from tissue and plasma obtained from three phase III clinical trials, each utilizing different testing methods, showed concordance rates of 70–83%, while in the BOLERO2 study, a higher concordance was observed for metastatic lesions (82%). *PIK3CA* mutations were more frequently detected in tissue samples than in liquid biopsies in both studies. Circulating tumor cells (CTC) have been also investigated as a possible source of biologic materials for the detection of *PIK3CA* mutations. An ultra-sensitive (0.05%) combination of allele-specific, asymmetric rapid PCR and melting analysis to detect hotspot mutations (exons 9 and 20) has been applied on the CTC of patients with breast cancer (67). In the present study, we were able to detect *PIK3CA* mutations in 35.1% (20/57) and 19.5% (23/118) of MBC and early breast cancers, respectively. Notably, no false-positive results were reported in healthy donors. Unsurprisingly, CTCs were isolated with a higher frequency in the metastatic setting, where the presence of *PIK3CA* mutations correlated with a worse outcome (67). A more recent study showed a comparison between ctDNA and CTC analysis for *PIK3CA* molecular assessment (68). By using the same ultra-sensitive approach, they observed a superior concordance rate among the two methodologies in MBC.

Taken together, these data show that the technology of liquid biopsy for ctDNA analysis has been successfully used in patients with breast cancer to identify *PIK3CA* alterations without additional invasive testing. The main advantages of this procedure compared to conventional tissue biopsy are the non-invasive nature and repeatability. These allow for a timely follow-up, potentially overcoming spatial and temporal heterogeneity of tumor (63). On the other hand, a major limitation of this approach is the lower concentration of tumor-derived DNA compared to traditional tissue specimens. For this reason, careful attention should be paid to the technology chosen for liquid biopsy molecular analyses (69).

## Conclusion

**Figure 3:**
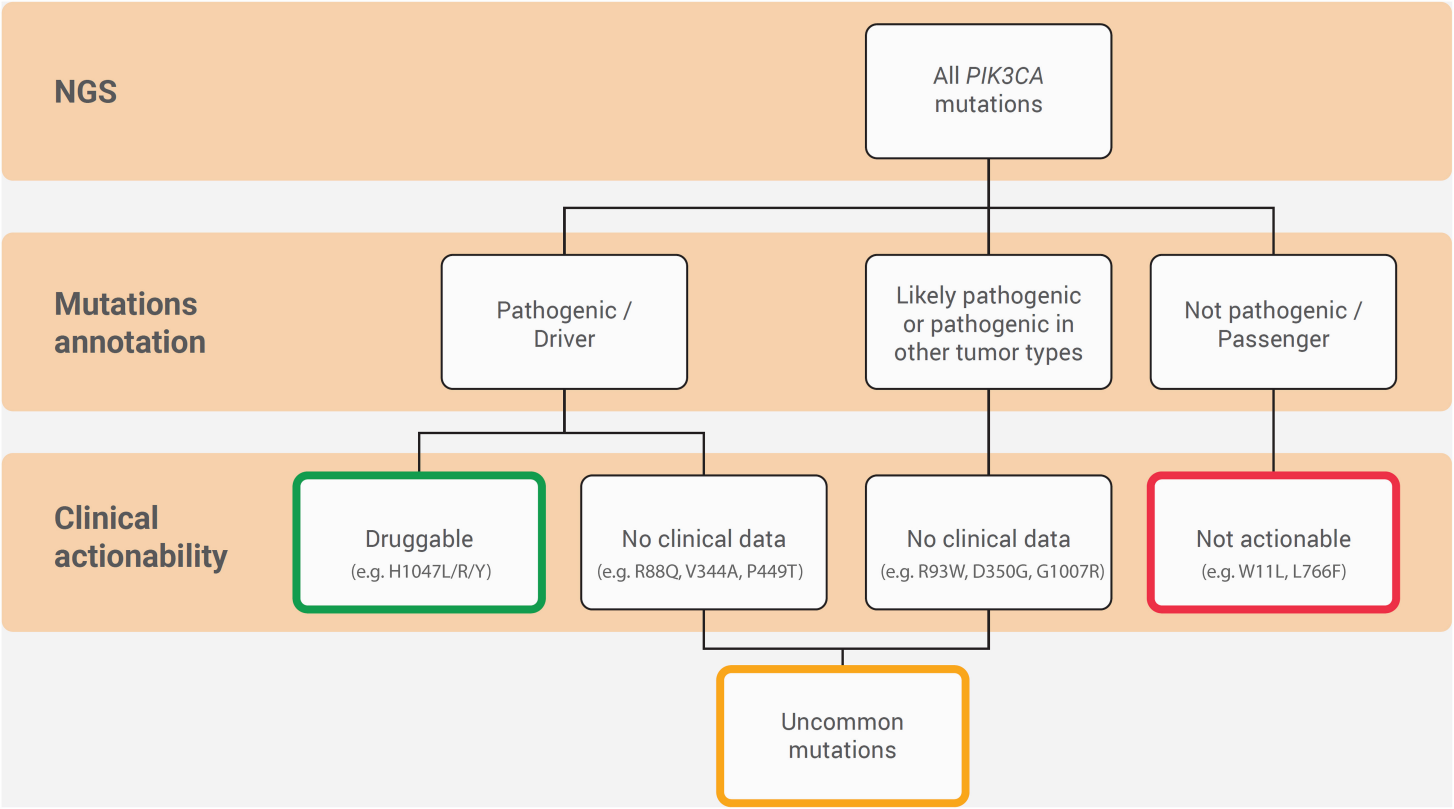
Hierarchy diagram portraying the annotation and clinical actionability of *PIK3CA* mutations in ER^+^/HER2^−^ metastatic breast cancer. The connecting lines indicate the relationship between them.

**Figure 4:**
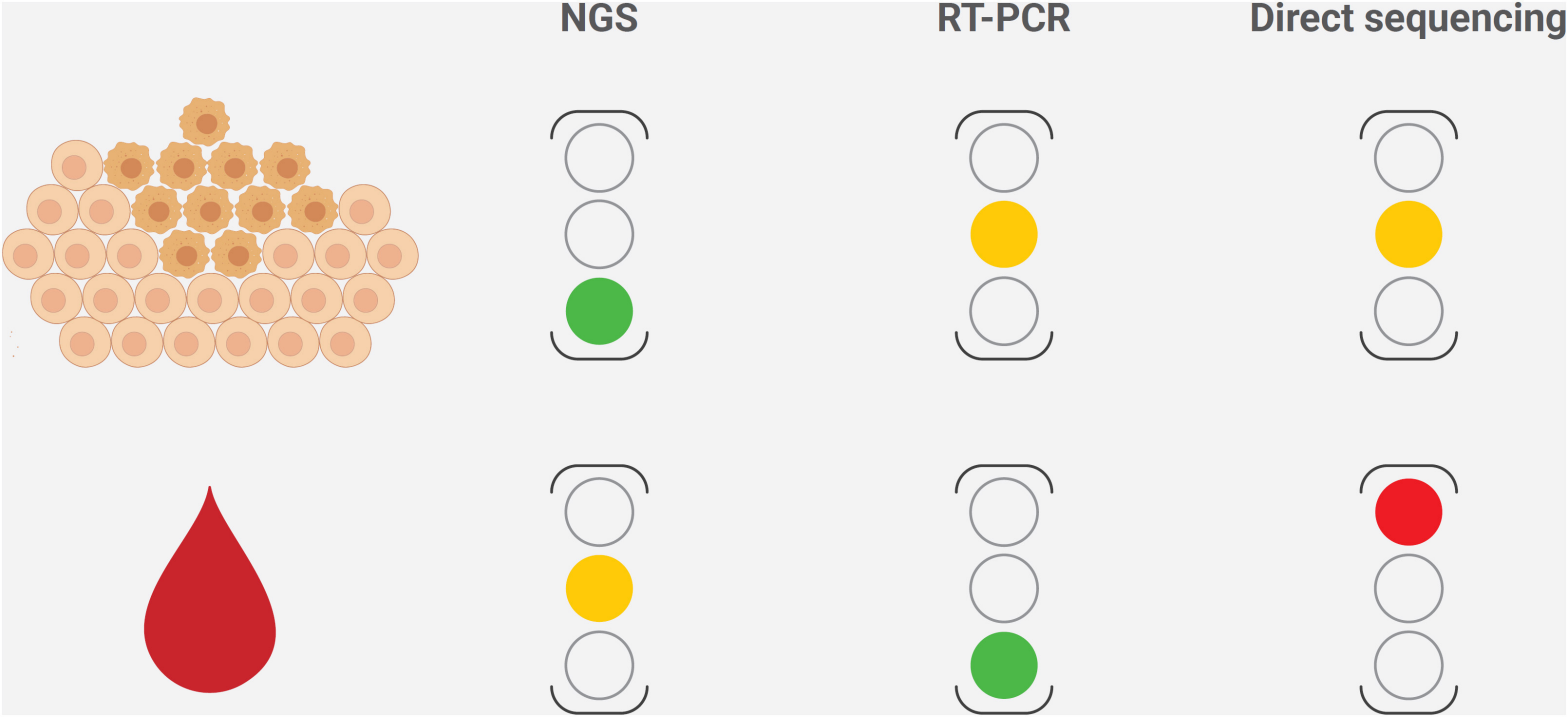
Strengths and limitations of the different methods to characterize *PIK3CA* mutational status in breast cancer, according to the sample type. The level of caution for each method is reported on the right, where the green light represents to proceed, the amber light represents warning on possible fails or false-negative results, and the red light represents discouraging the use of the technology in that setting. NGS, next-generation sequencing.

*PIK3CA* mutations are highly represented in ER^+^/HER2^−^ breast cancer and are of clinical interest due to the availability of targeted therapy with alpelisib in the metastatic setting (Figure 3). From the diagnostic perspective, it is crucial to adopt a tailored methodology to cover all of the clinically relevant gene alterations in the different clinical settings (Figure 4). The versatility and efficiency of RT-PCR, coupled with its cost-effectiveness, sensitivity, and specificity, facilitate the adoption of this technology in virtually all molecular pathology laboratory settings. Furthermore, this approach provides results in ultra-fast time, as the average duration of the reaction is between 30 min and 2 h. Although RT-PCR has several advantages over direct sequencing, the technology has also intrinsic limitations, including its limited multiplexing capability. Another important limitation of RT-PCR is the lack of kits and standardized protocols to detect all possible *PIK3CA* alterations (Figure 2). Furthermore, the vast majority of commercially available kits, including the FDA-approved RT-PCR test for *PIK3CA* mutational analysis, do not provide quantitative information about mutant allele frequency. On the other hand, NGS panels allow covering several different alterations simultaneously, even starting from low input of DNA, and provide information on the fraction of alleles carrying the mutation. Although the approximate costs of this technology, which requires specialized centers with highly trained personnel, are relatively high, the optimization of the laboratory workflow and volumes allows for a favorable cost-benefit ratio. Although tissue samples remain the most suitable material to be tested, other sources of DNA (i.e., liquid biopsies) may be used to analyze clinically relevant gene alterations. The latter may be particularly valuable whenever the tissue material is not available or is inaccessible. On the other hand, clinicians should be fully aware of the indications and limitations of this type of analysis in breast cancer. It is important to remark that in case of a negative result in the ctDNA analysis, a second liquid biopsy or, if feasible, a tissue biopsy should be analyzed to avoid false-negative results. Finally, though tissue samples from initial diagnosis are a reliable source for *PIK3CA* testing, it is generally preferable (if clinically feasible) to run the test on a biopsy of the metastatic site, as this may best reflect the actual genomic profile of the disease.

## Author Contributions

GV, EM, and AD: supervision. NF and UM: manuscript writing (first draft) and iconography. MF, CM, SB, SZ, CC, and PV: first draft revision. All authors: study design, revision, and approval of the final draft.

## Conflict of Interest

NF has received honoraria for consulting, advisory role, and/or speaker bureau from Merck Sharp & Dohme (MSD), Boehringer Ingelheim, and Novartis. UM received personal fees (for service in the speaker bureau and as an advisor) from Boehringer Ingelheim, AstraZeneca, Roche, MSD, Amgen, Merck, Eli Lilly, Thermofisher, and Diaceutics. MF received honoraria for consulting, advisory role, speaker bureau, and/or research funding from Astellas Pharma, QED Therapeutics, Diaceutics, Tesaro, Roche, Eli Lilly, and Novartis. CM received honoraria for consulting from Roche, Bayer, AstraZeneca, and Daiichi Sankyo. SB received honoraria for consulting/advisory role/speaker bureau from AstraZeneca, Novartis, Bayer, and Diaceutics. SZ received research funding from AstraZeneca. CC received honoraria for consulting/advisory role/speaker bureau from Novartis, Eli-Lilly, Pfizer, and Roche. PV received honoraria from AstraZeneca, BMS, Celgene, Eli-Lilly, GSK, Incyte, MSD, Novartis, Pfizer, and Teva and research funds from Novartis and Pfizer. AD received honoraria for advisory board roles from Bayer, Roche, Merck, PharmaMar, and Novartis Oncology. EM received honoraria for consulting, advisory role, and/or speaker bureau from Novartis, Roche, and Shire. GV received honoraria for consulting, advisory role, speaker bureau, travel, accommodation, expenses, and/or research funding from MSD Oncology, Pfizer, Dako, Roche/Genetech, Astellas Pharma, Novartis, Bayer, Daiichi Sankyo, Menarini, Ventana Medical Systems Dako/Agilent Technologies, Cepheid, and Celgene. The handling editor declared a shared affiliation, though no other collaboration, with one of the authors UM.
